# Retrobulbar hematoma following the repair of an orbital wall fracture: a case series

**DOI:** 10.1186/s40902-021-00289-4

**Published:** 2021-02-24

**Authors:** Jeong-Mo Kim

**Affiliations:** Department of Oral and Maxillofacial Surgery, Kangwon National University Hospital, Chuncheon, Korea

**Keywords:** Retrobulbar hematoma, Orbital wall fracture

## Abstract

**Background:**

Retrobulbar hematoma is a rare complication after the repair of an orbital wall fracture, but the caution is required because the condition can cause blindness.

**Case presentation:**

In this article, 3 cases of retrobulbar hematoma after the surgical repair of an orbital wall fracture are reported. In the first patient, the permanent loss of vision was involved, while in the second patient, the author was able to prevent loss of vision by performing immediate decompression after definite diagnosis and consulting with an ophthalmologist. In the third patient, there was no surgical treatment involved; he recovered on his own without major sequelae.

**Conclusions:**

Retrobulbar hematoma is a very serious condition that can result in blindness. Thus, when it is recognized, every effort should be made to preserve the patient’s vision and prevent blindness.

## Background

Retrobulbar hematoma (RBH), which can occur after the repair of an orbital wall fracture, is a rare but serious condition that can result in permanent loss of vision. Retrobulbar hematoma seems to result from bleeding caused by damage to the infra-orbital arteries or the anterior and posterior ethmoidal arteries during surgery. As bleeding continues, compression occurs within the enclosed retrobulbar space and leads to an increase in intraorbital pressure that can result in an occlusion of the central retinal artery (CRAO), ultimately causing an ischemic optic neuropathy that manifests as the loss of vision [1–5]. Girotto et al. [6] reported a 0.32% prevalence of retrobulbar hematoma (4/1240) over 11 years, and in Korea, Cheon et al. [4] reported a 0.17% prevalence (2/1180) over 6 years. In author’s case, from 2011 to 2019, a total of 261 cases of surgery of orbital wall repair were performed, and a total of 3 cases of retrobulbar hematoma were encountered (1.15%). Among these 3 cases, one involved the permanent loss of vision. In another case, author was able to prevent the loss of vision by performing immediate decompression after definite diagnosis. The remaining case healed without special treatment. Herein, author report and analyze these cases and review the literature.

### Cases presentation

#### Case 1

**Fig. 1:**
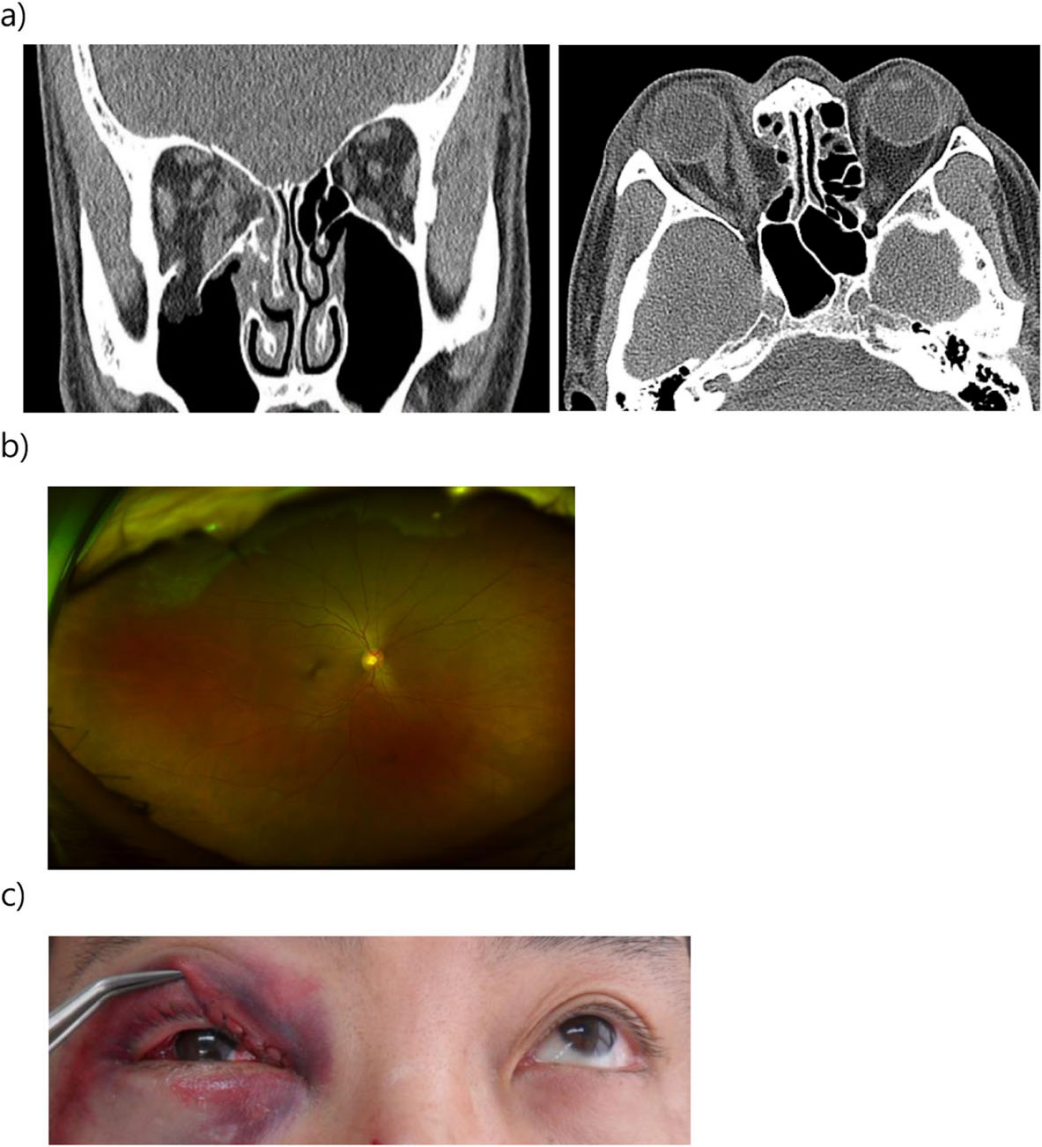
Preoperative CT images, fundus photograph, and eyelid photo. **a** Coronal and axial section showing fracture of the right superior/inferior/medial orbital wall and entrapment of the right inferior rectus muscle with orbital fat herniation to the right maxillary antrum. **b** Fundus photograph showing non-specific sign. **c** The limitation of the upward gaze is demonstrated

**Fig. 2:**
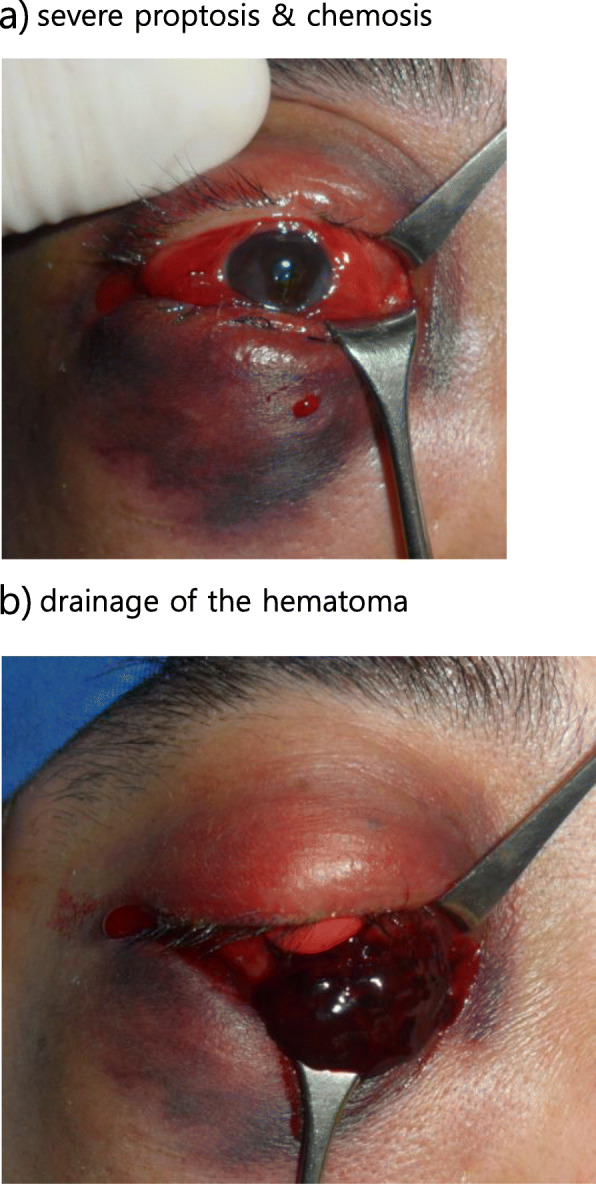
Eyelid photos. **a** Severe proptosis and chemosis. **b** Drainage of the hematoma

**Fig. 3:**
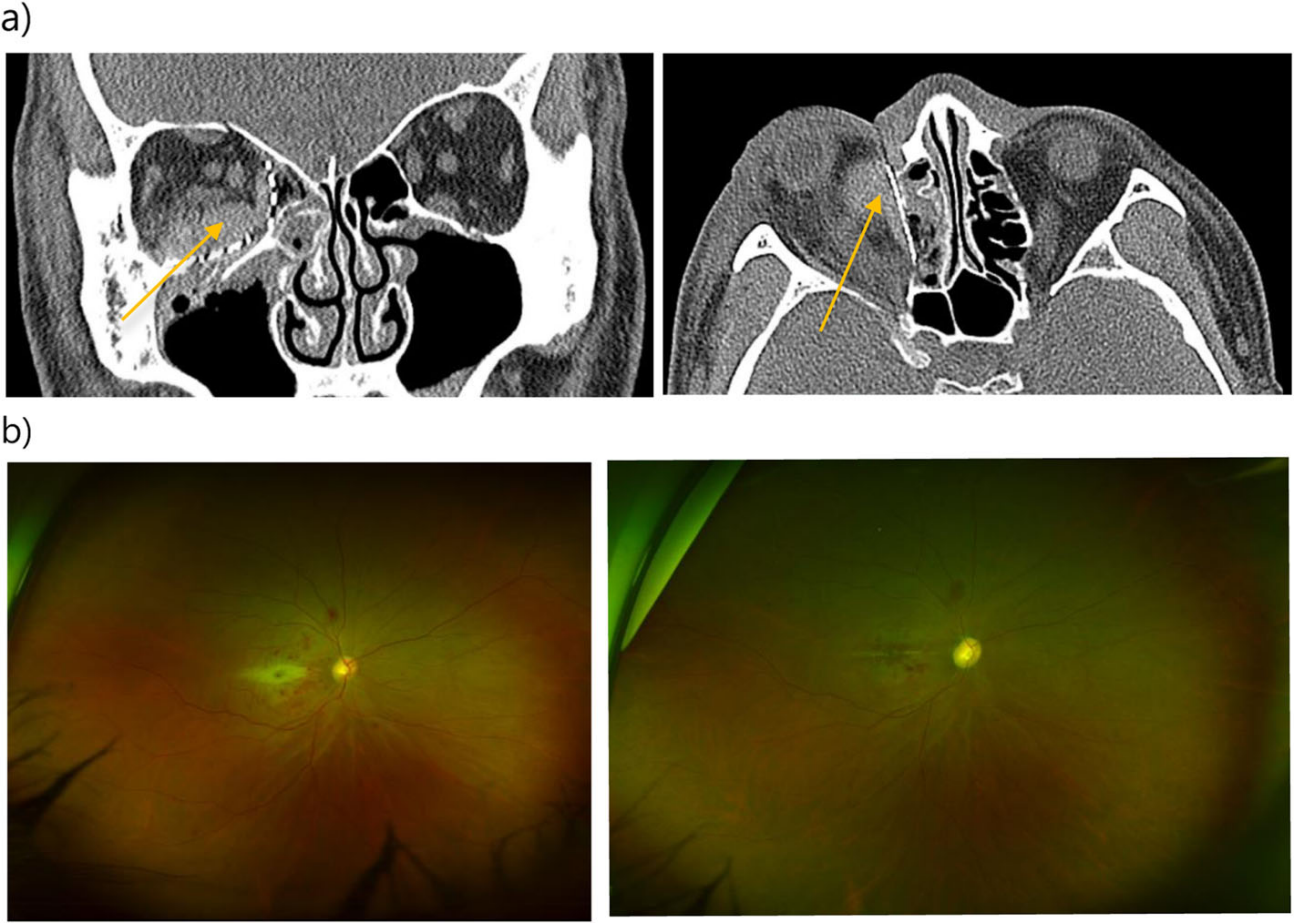
Postoperative CT images and fundus photographs. **a** Postoperative CT images of coronal and axial sections. The right medial and inferior orbital walls were reconstructed, and the med-por was properly located. However, a soft tissue density lesion was founded in left extraconal space along the medial and inferior orbital wall, indicating a retrobulbar hematoma (yellow arrow). **b** Left: fundus photograph of the right eye showing a cherry-red spot, a milky-white lesion on macula, and mild venous dilation. Right: 1-month after the previous fundus photograph

A 47-year-old male was involved in a traffic accident while driving in a car. Immediately after the accident, he visited our emergency room, and primary closure was performed. He was admitted to the Department of Oral & Maxillofacial Surgery for surgery of the orbital floor and medial wall fracture. His right eyelid had a wound of approximately 2 cm and was severely swollen so that his right eyeball was barely visible. Subconjunctival hemorrhage was present, and his vision was somewhat reduced. In the preoperative ophthalmologic examination, his visual acuity was 0.2 in the right eye and 1.0 in the left. There was a limitation of eye movement upward, and the patient experienced diplopia when looking forward and upward. Computed tomography revealed fracture of the right inferior and medial orbital wall and entrapment of the inferior rectus muscle with orbital fat herniation (Fig. 1). Six days later, the surgery was performed, with reconstruction of the orbital wall. On the day of surgery, there was no specificity. However, at dawn on the day after surgery, the patient complained of severe pain and proptosis in the right eye and no vision. Retrobulbar hematoma was confirmed on CT, and the patient was consulted to ophthalmology. The intraocular pressure of the right eye did not appear to be high; thus, lateral cantholysis was not performed. The patient was followed-up with, and he was treated with systemic corticosteroids in a standard dosage for 3 days (dexamethasone disodium phosphate, 15 mg/day). However, his vision did not recover, and on the third postoperative day, the access was reopened, and hematoma removal was performed (Fig. 2). One week later, his vision had not recovered, and ophthalmologic examination revealed blindness due to central retinal artery occlusion. The right fundus could be seen and showed a red spot, and white edema appeared to be caused by a complete CRAO. One month later, the optic disc appeared pale and atrophic (Fig. 3).

#### Case 2

**Fig. 4:**
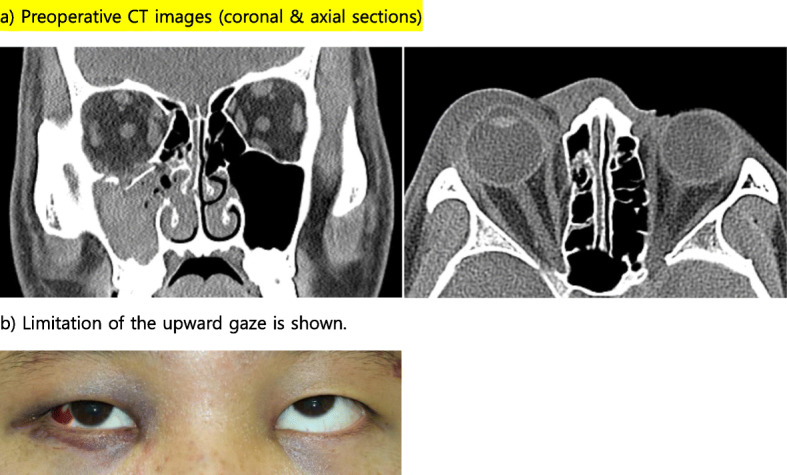
**a** Preoperative CT images (coronal and axial sections). **b** Limitation of the upward gaze is shown

**Fig. 5:**
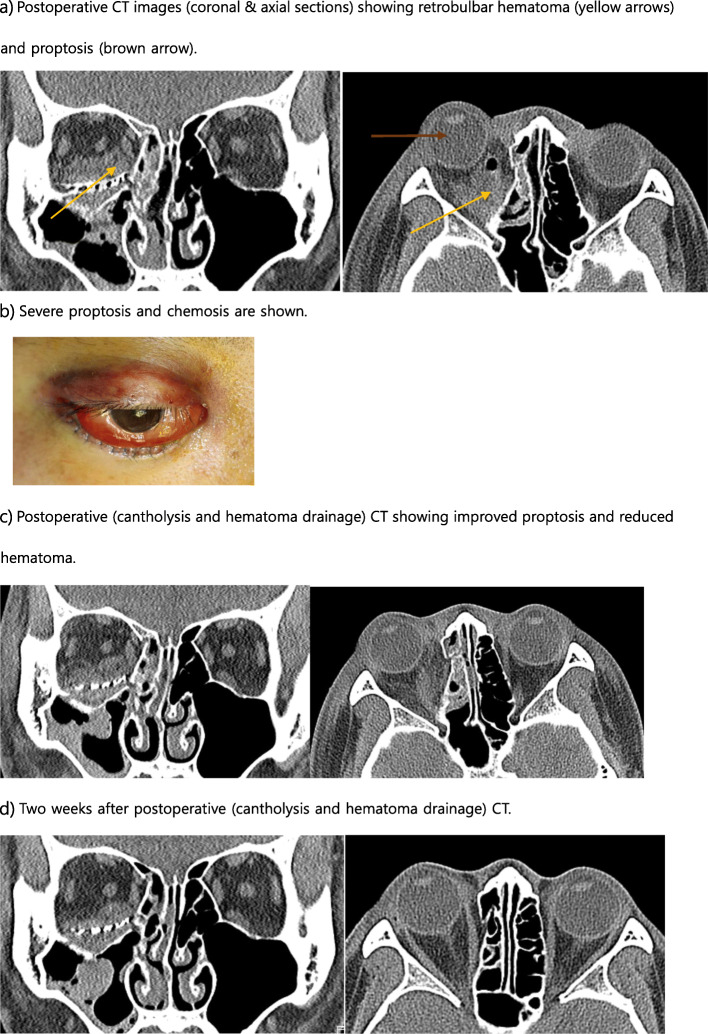
**a** Postoperative CT images (coronal and axial sections) showing retrobulbar hematoma (yellow arrows) and proptosis (brown arrow). **b** Severe proptosis and chemosis are shown. **c** Postoperative (cantholysis and hematoma drainage) CT showing improved proptosis and reduced hematoma. **d** Two weeks after postoperative (cantholysis and hematoma drainage) CT

**Fig. 6:**
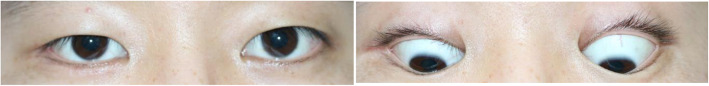
The inconspicuous scar and mild downward gaze limitation

A 21-year-old male was assaulted in the face, visited a hospital in another area and was diagnosed with orbital and nasal bone fractures (Fig. 4). After diagnosis, he visited the Oral and Maxillofacial Surgery of Kangwon-University Hospital the next day and was hospitalized for surgery. The patient had limited ocular movements and diplopia with upward and downward gaze, along with facial sensory impairment and nasal deformity. His visual acuity evaluated by preoperative ophthalmology was Rt. 0.3/Lt. 0.3. Two days after the injury, orbital wall reconstruction and nasal bone repair were performed under general anesthesia. On the 1st day, there was slight swelling, chemosis, and mild pain at the surgical site. The diplopia was almost improved, and there were no significant problems with his vision. The damaged skin sensation had also improved significantly. Therefore, corticosteroids were administered, and the patient’s course was monitored (dexamethasone 15 mg/day). On the second day of surgery, the patient complained of severe ocular pain and exhibited severe ocular protrusion. His vision also began to deteriorate rapidly, and ophthalmology asked for an evaluation. The CT showed retrobulbar hematoma (Fig. 5). The patient’s right eye visual acuity decreased to 0.01, and the intraocular pressure was elevated. Lateral canthotomy and cantholysis were immediately performed to reduce the intraocular pressure, and the hematoma was removed under general anesthesia. The next day, the patient’s pain was significantly decreased, and chemosis was decreased, but diplopia was observed when the patient gazed downward. The visual acuity was 0.04. After that, the pain was almost improved. After 1 week, the patient’s visual acuity improved to 0.2, and the corrected visual acuity was 0.7. Three weeks after surgery, his visual acuity was improved to 0.3. At 7 weeks after surgery, both eyes were corrected to 1.2, and the diplopia was improved. At 6 months after surgery, his visual acuity was normal, and the scar was not noticeable; however, mild diplopia when gazing downward was still present (Fig. 6).

#### Case 3

**Fig. 7:**
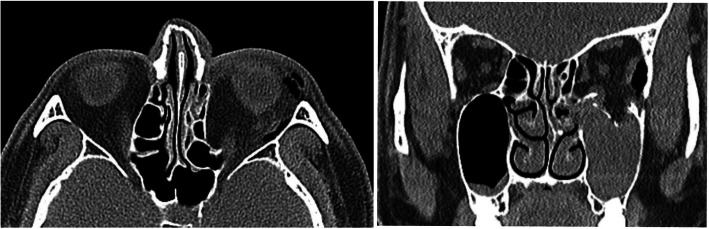
Preoperative CT (axial and coronal sections)

**Fig. 8:**
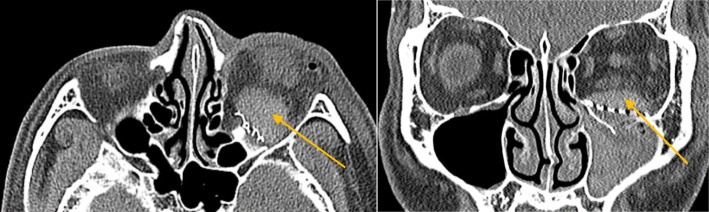
Postoperative CT (axial and coronal sections) showing retrobulbar hematoma (yellow arrows)

A 57-year-old male was injured in the face when falling down while working on a 3-m-high roof and immediately visited our emergency room. The patient exhibited limited ocular movement and diplopia when gazing upward, downward, and in both lateral directions, and he was examined and diagnosed with left orbital floor and medial wall fracture (Fig. 7). He was admitted to the Oral and Maxillofacial Surgery for orbital wall repair. The patient’s visual acuity evaluated by preoperative ophthalmology was measured at Rt. 1.2/Lt. 1.5. Orbital wall reconstruction was performed 5 days after injury. On day 1, there was no pain and mild chemosis, his eye movements returned to normal, and mild diplopia remained when gazing downward. Postoperative CT revealed retrobulbar hematoma (Fig. 8); corticosteroids were administered, and the patient’s course was monitored. At 2 days after surgery, the diplopia interval became narrower, and after 1 week, normal visual acuity was measured at 1.2/1.5. After 2 months, the diplopia was fully recovered, and the patient’s visual acuity was normal.

## Discussion

Retrobulbar hematoma following the repair of the orbital wall is a rare complication but requires considerable caution because it can cause visual impairment or blindness due to ischemic optic nerve injury or occlusion of the central retinal artery through increased intraocular pressure [6, 7].

When retrobulbar hematoma occurs, patients present with a variety of signs and symptoms, including circumbulbar pain, reduced visual acuity, blindness, proptosis, pupillary abnormalities, reduced extraocular movements, and increased intraocular pressure. In addition, nausea, vomiting, and headache may also be present [1–7]. Therefore, if such symptoms appear after surgery, it is important to confirm the presence of hematoma by CT scan, and the patient must be referred to an ophthalmologist to check their visual acuity and intraocular pressure.

If CT scans show hematoma and sudden visual impairment, active medical or surgical decompression should be considered to prevent the loss of vision and to recover any reductions in vision as soon as possible [4–6].

In the first case, the central retinal artery was occluded due to postoperative retrobulbar hemorrhage, leading to blindness. The next morning after surgery, the patient developed severe pain, proptosis, and decreased visual acuity. It seems that the bleeding had already advanced and the retinal arteries had begun to compress. In the intraocular pressure test, the patient’s intraocular pressure was not high; thus, it was decided to observe his progress. However, the patient’s impaired vision did not recover, eventually leading to blindness. Therefore, the time of surgical treatment was missed, and as a result, the patient missed their chance for visual recovery.

A central retinal artery occlusion is a very rare complication after surgery of the orbital fracture and is thought to be due to elevated intraocular pressure, mechanical stress, or optic nerve injury [7]. In this patient, reconstruction of a large orbital wall defect was performed through a transcaruncular approach with relatively poor visual access, and it is thought that there was a considerable pressure on the eyeball during this process. Postoperative bleeding developed into the retrobulbar hematoma, which led to elevated intraocular pressure and optic nerve atrophy. In addition, the effort to completely cover the fracture site may have impeded drainage of the hematoma into the sinus. Therefore, the intraorbital pressure could not be reduced, which is thought to have resulted in central retinal artery occlusion.

Irreversible ischemic damage often occurs within 60 min, and certainly within 2 h; thus, ischemia lasting for just 60–120 min can lead to permanent visual loss [6, 8–11]. Therefore, it is thought that rapid diagnosis and surgical decompression should have been made within at least 2 h.

The second case exhibited severe pain, proptosis, decreased visual acuity, and increased intraocular pressure from the second day of surgery. After the intraocular pressure was checked, lateral canthotomy and cantholysis were performed immediately by the ophthalmologist, and the patient’s intraocular pressure began to drop immediately. Additionally, the access was reopened, and the hematoma was removed under general anesthesia. Compared with the first case, it was thought that aggressive treatment could minimize the sequelae.

Lateral canthotomy and cantholysis are regarded as the treatment of first choice, as an effective method of orbital decompression for sight-threatening acute retrobulbar hemorrhage. This procedure is relatively simple and can be carried out in an outpatient or emergency room setting, under local anesthesia by any member of the medical staff [12, 13]. It is less clear whether more aggressive management of the source of the hemorrhage and hematoma is needed. Colletti et al. stated that if immediate symptom relief is not seen, one must proceed with open exploration of the orbit in search of the source of the bleed and remove the hematoma [1].

The third case showed a significant amount of retrobulbar hematoma and exophthalmos on the day after surgery, but the pain was not severe, and the patient exhibited normal vision. Therefore, corticosteroid in a standard dosage was administered, and the patient’s progress was observed. No visual loss or other complications occurred.

There are several guidelines for the treatment of retrobulbar hematoma. Many investigators insist on surgical decompression of the intraorbital region within a short time, but some investigators have reported good results with medical therapy alone [5, 14]. In addition, some authors have reported that medical therapy was not at all effective, while others have reported that the patients recovered by themselves without any treatment [15, 16].

Christie et al. analyzed 16 articles and 93 cases of retrobulbar hematoma and reported that time to treatment had a robust correlation with outcomes, which were extremely time-sensitive. A shorter time to treatment was associated with a greater likelihood of full recovery and less likelihood of blindness. While the addition of steroids to surgery was not found to be statistically significant for improving visual outcomes, the overall intervention of steroids (with or without surgery) trended towards an improvement in visual outcomes [5].

Finally, to prevent retrobulbar hematoma, care should be taken to control bleeding during surgery, and excessive retraction should be avoided. After surgery, an icepack should be applied to reduce swelling and bleeding. Procedures that can increase intraocular pressure should be avoided. For example, valsalva manipulation, which would apply excessive force during sneezing, coughing, nose blowing, waist bending, vomiting, or bowel movement, should be avoided.

## Conclusion

If symptoms of eyeball pain, proptosis and a sudden drop in vision present following intraorbital surgery and retrobulbar hematoma are suspected, the condition must be recognized as an emergency. The patient should be diagnosed rapidly, the intraocular pressure and visual acuity should be checked, the hematoma should be decompressed immediately, and every effort should be made to preserve visual acuity and to prevent blindness.

## Data Availability

None.

## Abbreviations

CRAO: Central retinal artery occlusion
CT: Computed tomography
RBH: Retrobulbar hematoma
